# Characterization, Microbial Community Structure, and Pathogen Occurrence in Two Typical Eel Farms

**DOI:** 10.3390/microorganisms13071624

**Published:** 2025-07-10

**Authors:** Jing-Ying Lai, Hui-Rong Lin, Xiao-Hui Sun, Gong-Ren Hu, Rui-Lian Yu, Jia-Qi Li

**Affiliations:** College of Chemical Engineering, Huaqiao University, Xiamen 361021, China; jylaifj@163.com (J.-Y.L.); grhu@hqu.edu.cn (G.-R.H.); ruiliany@hqu.edu.cn (R.-L.Y.); ljq1912669203@163.com (J.-Q.L.)

**Keywords:** nitrogen pollution, aquaculture wastewater treatment, pathogens, process optimization

## Abstract

Pollutants and pathogens in aquaculture systems may cause economic losses and threaten public health. Yet, the risks associated with microbiological contaminants and their relationship with environmental factors remain largely unknown. In this study, two typical eel farms in southeast China were chosen for investigation of water quality and microbial community in the treatment processes. It was found that flocculant addition can only effectively reduce total phosphorus (TP) in both farms. However, excessive total nitrogen (TN) was found (6.16 mg/L and 6.74 mg/L, respectively). NH_4_^+^ (3.98 mg/L) was the main nitrogen pollutant in QR farm, while NO_3_^−^ (3.81 mg/L) and NO_2_^−^ (1.22 mg/L) were the main nitrogen pollutants in ZJ farm. The treatment processes could not effectively remove nitrogen pollution, and the abundance of nitrogen functional bacteria was low. NO_2_^−^ was positively correlated with Verrucomicrobiota (*p* < 0.05). NH_4_^+^ and TN were significantly negatively correlated with Nitrospirota and *unclassified_f_Anaerolineaceae*, respectively (*p* < 0.05). Some typical pathogens associated with aquaculture (e.g., *Lactococcus*) and human beings (e.g., *Escherichia-Shigella*,) were found in the systems. This study proposes suggestions for aquaculture tailwater by analyzing the shortcomings of the existing treatment processes. Meanwhile, it offers certain support for the prevention of pathogen risks in aquaculture systems.

## 1. Introduction

In recent years, global aquaculture production has been continuously increasing. Since the 1990s, China has been the largest fishery production region [1]. Fujian Province, located in southeast China, is one of the coastal provinces developing fisheries because of its rich water resources and mudflats. Aquaculture methods are diversified in Fujian, mainly including land-based facility aquaculture, shallow sea aquaculture, deep-sea aquaculture, and rice field aquaculture. A series of pollutants, including nitrogen pollutants, phosphates, pathogens, as well as other harmful substances, such as feces and residual feeds, can be found in aquaculture systems [2].

The accumulation of waste, such as excrement and residual feeds, leads to an increase in particulate matter and nutrients in water, which can have adverse effects on the health of aquaculture organisms and lead to the deterioration of the surrounding water bodies if these pollutants are not treated well [3,4]. Nitrogen pollutants in aquaculture systems mainly include ammonia nitrogen (NH_4_^+^), nitrite nitrogen (NO_2_^−^), and nitrate nitrogen (NO_3_^−^) [5]. NH_4_^+^ pollutants can lead to damage to the endocrine and liver systems and affect the respiratory system of aquaculture organisms [6,7]. Excessive phosphate pollutants can lead to eutrophication and endanger aquaculture organisms [8]. At the same time, the presence of pathogens may cause diseases in farmed animals and may also pose potential risks to human health and the ecological environment, such as cholera, diarrhea, and respiratory diseases [9]. So, it is necessary to investigate microbiological contaminants, especially the abundance of pathogens in aquaculture systems.

In order to improve water quality in aquaculture systems, different technologies have been applied. Colloids and small suspended pollutants can be settled using flocculants. However, they usually cause secondary pollution [10]. Chemical treatment costs are high [11]. Physical methods predominantly focus on the mitigation of symptoms, rather than addressing the root etiological factors of pollution, thereby leading to the persistence and incomplete elimination of hazardous substances in the polluted water [12]. Biological methods have many advantages, such as good and efficient treatment effects, low cost, minimal secondary pollution, wide application range, and multiple sources of microbial agents. So, microbial technology is widely used in aquaculture wastewater treatment [13,14]. Biological activity and the abundance of functional microbes are deterministic factors driving pollutant transformation and degradation [15]. So, it is necessary to investigate microbial community structure, especially functional microorganisms in the aquaculture treatment process.

In this study, two typical eel farms with high requirements for the quality of aquaculture water in Nanping City, Fujian Province, were selected to explore the water quality and the microbial community structure characteristics in the aquaculture systems. By analyzing water quality and the abundance of functional microbes and pathogens, as well as measuring the relationship between microbial communities and environmental factors, we aim to provide information for potential risk prevention, point out the shortcomings of existing treatment processes, and put forward improvement measures.

## 2. Materials and Methods

### 2.1. Overview of the Study Area and Sampling Locations

Nanping City (26°30′–28°20′ N, 117°00′–119°25′ E) is located in the upper reaches of the Minjiang River Basin. As a key water source conservation area, it plays an indispensable role in maintaining the ecological balance of the basin, with the proportion of Class I-II high-quality water exceeding 90%. These good-quality water resources provide a good water source guarantee for aquaculture. As of October 2020, there are a total of 102 eel farming enterprises in this city. The total area of eel farming has reached 3820 acres, with a farming scale of approximately 10,893 tons. The rapid development of aquaculture has brought pollution to the local surface water.

**Figure 1:**
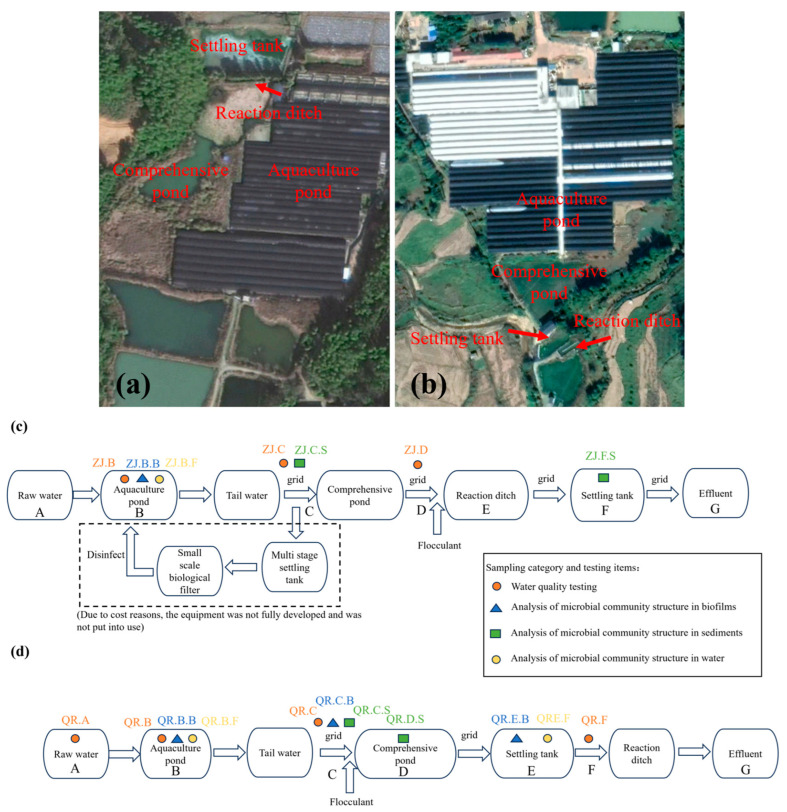
Satellite images and aquaculture water treatment processes of ZJ and QR: (**a**) ZJ aquaculture farm; (**b**) QR aquaculture farm; (**c**) the aquaculture water treatment process of ZJ; (**d**) the aquaculture water treatment process of QR.

ZJ farm and QR farm, located in Nanping City, are typical large-scale centralized eel farms. Both farms use raw water of good quality. The treatment processes of adding flocculants in these two farms are shown in Figure 1. However, due to the limitation of operational costs, some treatment facilities are not in operation, as shown in Figure 1c. For the purpose of this work, aquaculture water (ZJ.B), tailwater (ZJ.C), comprehensive pond effluent (ZJ.D), biofilm on the pond wall (ZJ.B.B), sediment at the tailwater grid (ZJ.C.S), and settling tank sediment (ZJ.F.S) in ZJ farm and raw water (QR.A), aquaculture water (QR.B), biofilm on the pond wall (QR.B.B), tailwater (QR.C), biofilm and sediment at the tailwater grid (QR.C.B and QR.C.S), comprehensive pond sediment (QR.D.S), and settling tank biofilm and effluent (QR.E.B and QR.F) in QR farm were sampled. The biofilm samples of the aquaculture pond and settling tank were collected from the walls of the pond, and the biofilm samples at the tailwater grid were collected from the surface biofilm of the water. Samples for detecting the microbial community in water were obtained by filtering water samples through membrane filters (ZJ.B.F, QR.B.F, and QR.E.F). The sampling sites are shown in Figure 1c,d.

### 2.2. Water Quality Analysis

NH_4_^+^ was tested using Nessler’s reagent spectrophotometry according to HJ 535-2009 in China [16]. NO_3_^−^ determination was carried out using ultraviolet spectrophotometry in accordance with HJ/T 346-2007 [17]. The NO_2_^−^ test was carried out using spectrophotometry in compliance with GB 7493-87 [18]. Following the specifications of HJ 636-2012 [19], the TN was tested using alkaline potassium persulfate ultraviolet spectrophotometry. TP was tested using ammonium molybdate spectrophotometry per the requirements of GB 11893-89 [20].

### 2.3. DNA Extraction

The water samples were first filtered through 0.8 μm filter membranes to remove suspended solids and algae and then filtered through 0.22 μm filter membranes to separate microorganisms from the water. The filter membrane was then cut into pieces with sterile scissors for later use. Biofilm samples were collected by scraping with cotton swabs. Sediment samples were collected with sterile sampling shovels. Using the FastDNA spin kit for soil (MP Biomedicals, Santa Ana, CA, USA) DNA extraction kit, DNA was extracted from each sample according to the standard procedure described in the instructions. DNA concentration and purity were tested using microspectrophotometry (Nanodrop One (Thermo Fisher Scientific, Waltham, MA, USA)). The extracted DNA samples were stored in a −80 °C freezer and sent to Guangdong Magigene Co., Ltd. (Guangzhou, China) for 16S rRNA gene sequencing later.

### 2.4. Microbial Community Structure Analysis

The V3-V4 region of the bacteria 16S rRNA gene was amplified with primers 5′-ACTCCTACGGGAGGCAGCA-3′ and 5′-GGACTACHVGGGTWTCTAAT-3′ to investigate the microbial community structure. Thermal cycling consisted of initial denaturation at 94 °C for 5 min, followed by 30 cycles of 94 °C for 30 s, 52 °C for 30 s, and 72 °C for 30 s, followed by a final extension step of 72 °C for 10 min. The PCR products were purified and then sequenced using Illumina or MGI platforms (Guangdong Magigene Biotechnology Co., Ltd., Guangzhou, China). The original image data files obtained by sequencing were converted into Raw Reads by Base Calling analysis, which contained sequence information of Reads and their corresponding sequencing quality information. The original data was filtered by fastp (an ultra-fast all-in-one FASTQ preprocessor, version 0.14.1, https://github.com/OpenGene/fastp, accessed on 29 June 2025) and cutadapt (https://github.com/marcelm/cutadapt/, accessed on 29 June 2025) to obtain high-quality data and was then spliced using usearch-fastq_mergepairs (V10, http://www.drive5.com/usearch/, accessed on 29 June 2025) to obtain the Raw Tags. After that, fastp (version 0.14.1, https://github.com/OpenGene/fastp, accessed on 29 June 2025) was used to obtain the Clean Tags. The tags were clustered according to 97% similarity by the Uparse algorithm [21]. Tags with similarities greater than 97% were classified as Operational Taxonomic Units (OTUs).

### 2.5. Data Analysis

Statistical analysis was conducted using IBM SPSS software (V27). Intrinsic diversity analysis (i.e., alpha diversity) of each microbial sample was conducted by calculating species richness (Chao1 index) and the species diversity index (Simpson index) using usearch-alpha_div (V10, http://www.drive5.com/usearch/, accessed on 29 June 2025). Distance-based redundancy analysis (RDA) using R software (version 3.6.3) was used to study the environmental factors that affect microbial community structure among different samples. All tables and graphs were drawn using Excel 2022 and Origin 2022.

## 3. Results

### 3.1. Water Quality in the Treatment Processes

The water quality at each sampling site is shown in Table 1. Due to the high requirements for eel farming conditions, the water quality of the raw water is good. TN and TP concentrations at the grid of the concentrated discharge of aquaculture effluent from ZJ farm and QR farm are 6.16 ± 0.57 mg/L and 6.74 ± 0.11 mg/L, and 1.54 ± 0.02 mg/L and 1.27 ± 0.03 mg/L, respectively, which exceed the secondary discharge standards of TN ≤ 5 mg/L and TP ≤ 1.0 mg/L for freshwater pond aquaculture water discharge requirements (SC/T 9101-2007) [22] (Table S1). Nitrogen pollutants in QR farm are mainly NH_4_^+^ (3.98 ± 0.12 mg/L in the aquaculture effluent), while those in ZJ farm are mainly NO_3_^−^ and NO_2_^−^ (3.81 ± 0.11 mg/L and 1.220 ± 0.01 mg/L, respectively. in the aquaculture effluent). In QR farm, after using flocculants, the concentration of TP was significantly decreased to 0.04 mg/L (*p* < 0.05). The TP content in the effluent can meet the discharge standard (TP ≤ 1.0 mg/L).

### 3.2. Analysis of Microbial Community Composition and Diversity

#### 3.2.1. Analysis of Microorganisms at the Phylum and Genus Levels

**Figure 2:**
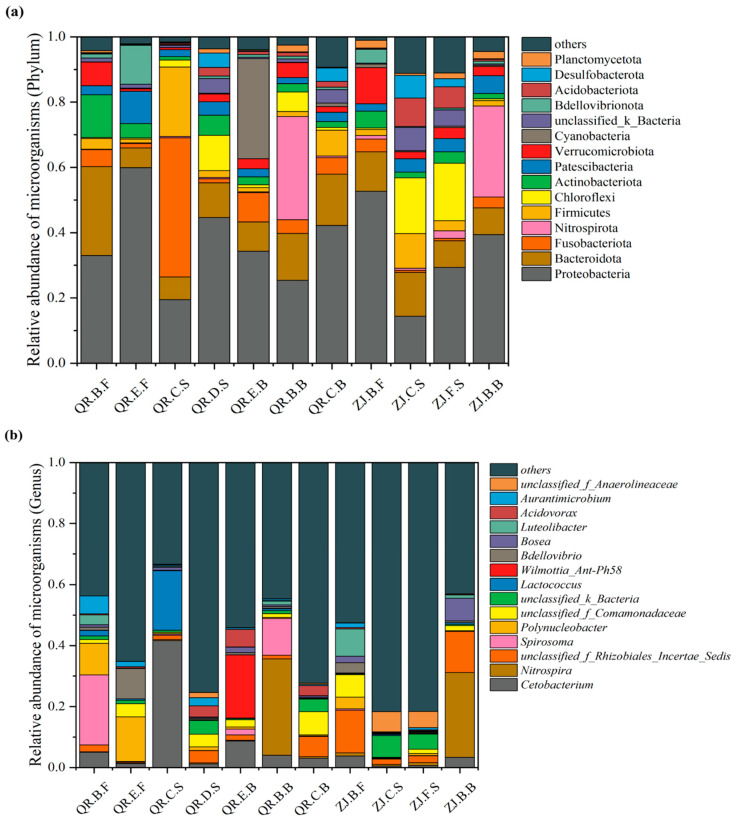
Relative abundance of microorganisms at the phylum (**a**) and genus (**b**) levels in ZJ and QR.

The abundance of the dominant microorganisms (phylums and genera) and microorganisms varies among samples of different loci and morphologies (Figure 2).

The dominant microorganisms at the phylum level are Proteobacteria, Bacteroidota, and Actinobacteria. Proteobacteria occupies the highest proportion in QR.B.F, QR.E.F, QR.D.S, QR.E.B, and QR.C.B and ZJ.B.F, ZJ.F.S, and ZJ.B.B., ranging from 29.33% to 59.89%. Chloroflexi, Proteobacteria, and Bacteroidota account for 17.05%, 14.35%, and 13.49%, respectively, in ZJ.C.S. Firmicutes account for 1.14–21.31% in both farms. The proportion of Nitrospirota in the pond walls of the two aquaculture farms is high, accounting for 31.62% and 27.86%, respectively. However, in the subsequent effluent treatment process, the proportion of Nitrospirota is relatively low. In addition to Proteobacteria, Cyanobacteria also account for 30.71% in QR.E.B.

At the genus level, the dominant genera are *Cetobacterium*, *Luteolibacter*, *Acidovorax*, *Bosea*, and *Nitrospira*. *Cetobacterium* is dominant in QR.C.S, with a relative abundance of 41.6%, while the proportion in other sites ranges from 0.52% to 8.69%. *Luteolibacter* and *Acidovorax* in QR farm are 0.055–3.2% and 0.12–5.78% and are 0.15–9.01% and 0.08–0.46% in ZJ farm. The relative abundance of *Bosea* in ZJ.B.B (7.43%) and ZJ.B.F (2.1%) is higher than that in other sites, but the highest abundance of *Bosea* in QR farm was in QR.E.B., accounting for 1.78%.

#### 3.2.2. Analysis of Microbial Community Diversity and Similarity

**Figure 3:**
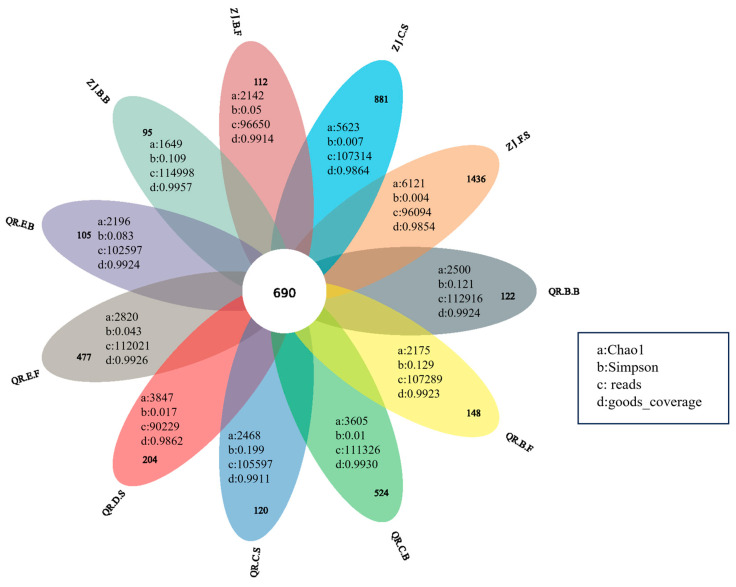
Shared and unique OTUs and the alpha diversity index of different positions.

OTUs of each site were obtained by sequencing the 16S rRNA gene. As shown in Figure 3, the goods_coverage of all samples is greater than 0.98, indicating that the sequencing results could cover most of the community, and most microorganisms had been detected, which could ensure the accuracy of the sequencing results. The number of OTUs shared by all sites is 690; ZJ.B.B and ZJ.F.S have the minimum (95) and maximum (1436) unique OTUs, respectively. The unique OTU number of the biofilm at the tailwater grid of QR farm is 524, which is the highest in QR farm. QR.E.F, QR.D.S, QR.B.F, QR.B.B, QR.C.S, and QR.E.B have 477, 204, 148, 122, 120, and 105 unique OTUs. ZJ.F.S has the highest richness and diversity, with a Chao1 index of 6121 and a Simpson index of 0.004. In QR farm, the species richness and diversity of D.S and C.B are high, with Chao1 indices of 3847 and 3605 and Simpson indices of 0.017 and 0.010, respectively. The areas with the highest species richness in both farms are in the sediment at the sites where the flocculants are adsorbed and settled.

#### 3.2.3. Abundance of Pathogenic Microorganisms

**Figure 4:**
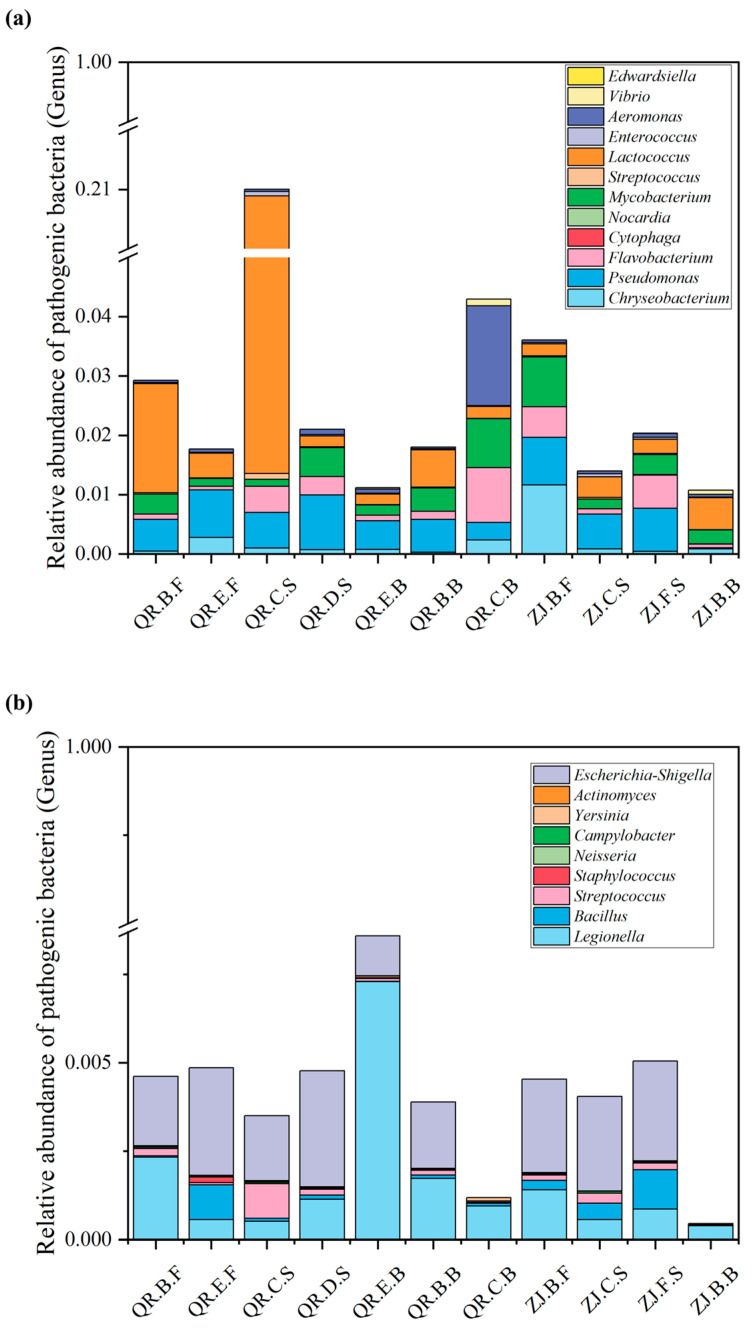
Relative abundance of aquaculture organisms pathogenic bacterial genus (**a**), and human pathogenic bacterial genus (**b**) in different locations of the two freshwater farms.

The relative abundances of main pathogens at each site of ZJ farm and QR farm are shown in Figure 4. As shown in Figure 4a, the abundance of pathogens associated with aquaculture organisms in ZJ farm is relatively low, ranging from 1.07% to 3.6%. The abundance of pathogens in the pond water is the highest, mainly including *Chryseobacterium*, *Pseudomonas*, and *Mycobacterium*. The abundance of pathogens related to the aquaculture organisms in QR farm ranges from 1.12% to 21%, with the highest relative abundance of pathogenic bacteria in the sediment at the tailwater grid, followed by biofilm at the tailwater grid and aquaculture pond water, mainly *Lactococcus* and *Pseudomonas*.

As for pathogenic bacteria associated with human beings (Figure 4b), in QR farm, the highest relative abundance was found in the biofilm of the settling tank, which was 0.86%. The site with the highest relative abundance in ZJ farm is the sediment of the settling tank, which is 0.51%. The vast majority of pathogenic bacterial genera are *Escherichia-Shigella* and *Legionella*.

### 3.3. Correlation Analysis with Environmental Factors

**Figure 5:**
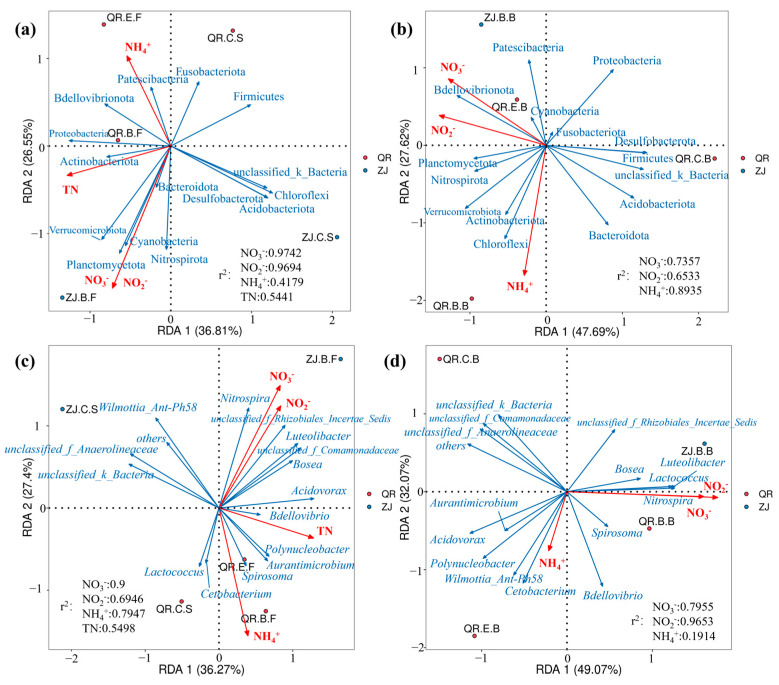
RDA analysis of environmental factors and microbial phylum and genus levels: (**a**) microbial phylum level in water and sediment; (**b**) microbial phylum level of biofilm; (**c**) microbial genus level in water bodies and sediment; (**d**) microbial genus level of biofilm.

RDA was used to investigate the effects of water quality (NH_4_^+^, NO_3_^−^, NO_2_^−^, TN) at different sites on the microbial community structure in water, sediment (Figure 5a,c), and biofilm (Figure 5b,d). In Figure 5a, eigenvalues of 36.81% for the x-axis and 26.55% for the y-axis accounted for 63.36% of the total variance. In Figure 5b, eigenvalues of 47.69% and 27.62% for the x- and y-axes, respectively, explained 75.31% of the total variance. Similarly, in Figure 5c, eigenvalues of 36.27% for the x-axis and 27.4% for the y-axis accounted for 63.67% of the total variance. In Figure 5d, eigenvalues of 49.07% and 32.07% for the x- and y-axes, respectively, explained 81.14% of the total variance. These results show that the main factors affecting the changes of microbial community in water are related to the nitrogen content of water, especially the concentration of NO_2_^−^ and NO_3_^−^. In water and sediment, NO_2_^−^ and NO_3_^−^ are significantly negatively correlated with microorganisms at the phylum level (*p* < 0.05). And, the coefficient of determination (r^2^) reflects the degree of the influence of environmental factors on microorganism distribution. The coefficients of determination of NO_2_^−^ and NO_3_^−^ are 0.9694 and 0.9742, respectively, indicating that NO_3_^−^ has a slightly greater impact on microorganism distribution at the phylum level than NO_2_^−^.

**Figure 6:**
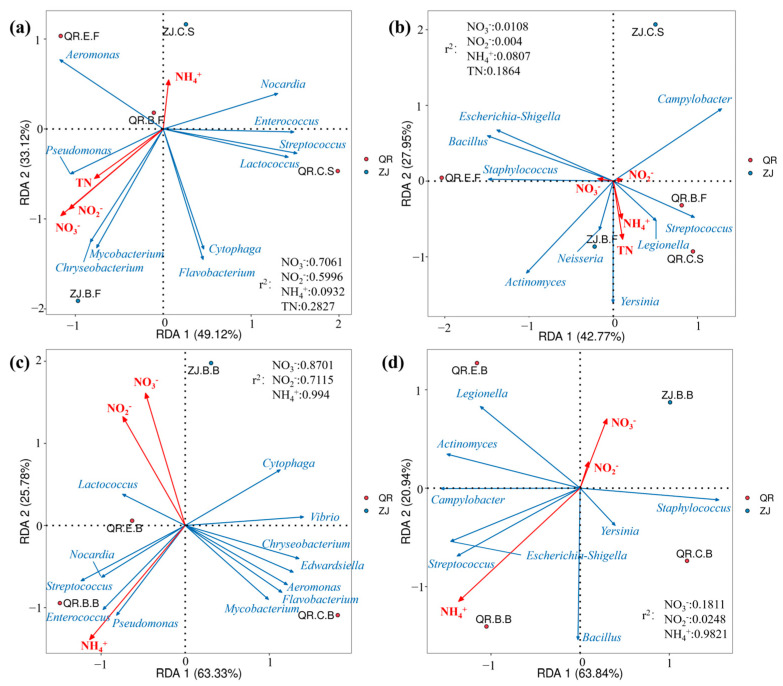
RDA analysis of environmental factors and pathogens: (**a**) pathogens associated with aquaculture organisms in water and sediment; (**b**) pathogens related to humans in water and sediment; (**c**) pathogens associated with aquaculture organisms in biofilm; (**d**) pathogens related to humans in biofilm.

Figure 6 shows the correlation between environmental factors and pathogens. The RDA analysis of environmental factors and pathogens associated with aquaculture organisms in water and sediment shows eigenvalues of 49.12% and 33.12% for the x- and y-axes, respectively, explaining 82.24% of the total variance. While the correlation between environmental factors and pathogens related to humans, eigenvalues of 42.77% for the x-axis and 27.95% for the y-axis accounted for 70.72% of the total variance. Similarly, the RDA analysis of environmental factors and pathogens associated with aquaculture organisms in biofilm indicates eigenvalues of 63.33% and 25.78% for the x- and y-axes, respectively, explaining 89.11% of the total variance. While the RDA between environmental factors and pathogens related to humans in biofilm, eigenvalues of 63.84% for the x-axis and 20.94% for the y-axis accounted for 84.78% of the total variance. In water and sediment, pathogens associated with aquaculture organisms and humans are mainly affected by NO_3_^−^ and TN, respectively. While in biofilm, pathogens are mainly affected by NH_4_^+^.

## 4. Discussion

### 4.1. Shortcomings of the Water Treatment Processes

The current tailwater treatment process in the two eel farms involves adding flocculants to adsorb and remove pollutants, as well as combining comprehensive pond and settling tanks to purify the tailwater. At the entrance of the reaction ditch in ZJ farm, flocculants were added. However, by comparing the water quality of the tailwater grid with the water quality before adding flocculants, it shows that the ecological regulation of the comprehensive pond alone has no significant effect on the removal of nitrogen and phosphorus. On the other hand, QR farm added flocculants after the tailwater grid. With the action of flocculants, TP concentration significantly decreased to 0.04 mg/L (*p* < 0.05), which can meet the discharge standard (TP ≤ 1.0 mg/L). However, after passing through the comprehensive pond and settling tank, the concentrations of NH_4_^+^ and TN increased compared with the water quality of the tailwater grid, indicating that the flocculant has a poor nitrogen removal effect. This may be due to the lush vegetation in the comprehensive pond of QR farm. Some nitrogen-fixing bacteria live in the rhizosphere, foliar, or tissue gaps of the plant host, using their own nitrogenase to fix nitrogen and using the products of plant photosynthesis as a carbon source, resulting in an increase in NH_4_^+^ or dissolved organic nitrogen concentration in the water [23].

There are also many residual feeds and excrements from breeding eels in the aquaculture water [24]. The effluent from the aquaculture pond is first filtered through the grid, where most of the suspended solids are trapped and enriched, which have high organic matter and nutrient content and are enriched with various bacteria. The high biodiversity of both aquaculture systems was localized to benthic substrates within flocculant deposition zones, where flocculant complexes undergo preferential sedimentation and microbial colonization. The sediment environment is rich in high concentrations of phosphorus and nitrogen adsorbed by flocculants in the water. And, the increase in nitrogen and phosphorus can promote microbial growth [25,26]. Flocculants can form polymers with phosphate, which form large flocs in water and precipitate by gravity. However, these polymers may re-release, thereby posing secondary contamination risks to downstream water quality.

In summary, the nitrogen and phosphorus concentrations in the tailwater of two eel farms in this city exceed the discharge standard. The current tailwater treatment process using flocculants is more effective in removing TP, but phosphorus removal flocculants will settle at the bottom of the pond. The sediment needs to be excavated and cleaned at intervals; otherwise, the pollutants will be re-released into the water body again. Anaerobic environments may lead to the re-release of phosphorus [27]. The NH_4_^+^ assimilated by algae in aquaculture farms may mineralize into NH_4_^+^ during the decay stage of algae, and the organic matter accumulated in sediments may also release nitrogen through mineralization [28,29]. At the same time, the current tailwater purification process has systematic deficiencies in nitrogen removal and cannot effectively remove nitrogen.

As for microorganisms in aquaculture systems, Proteobacteria, Bacteroidota, Chloroflexi, and Actinobacteriota exist in every site and are the majority of the microbial composition in both eel farms. They are common species in environmental samples [30]. Patescibacteria are typically present in both groundwater and surface water [31]. Compared with other sites, the relative abundance of Firmicutes in C.S is higher, which is 21.31% in QR farm and 10.62% in ZJ farm. Firmicutes are often detected in wastewater with high nutrient content (nitrogen and phosphorus). C.S is the sediment at the tailwater grid where large particles of residual bait and biological excreta are intercepted and precipitated. C.S in both farms is rich in nutrients. Nitrospirota is a common bacterial species involved in nitrogen cycling [32]. However, compared with an aquaculture pond, the proportion of Nitrospirota in the treatment process is relatively low. Acidobacteriota is involved in various biological denitrification processes, including partial nitrification, anaerobic ammonium oxidation, short-term nitrification, and denitrification, as well as nitrification denitrification. Acidobacteriota can utilize protons on the surface of cell membranes or metabolites as electron donors and carbon sources as electron acceptors in vivo under hypoxic conditions or use inorganic carbon sources (such as CO_2_, HCO_3_^−^, CO_3_^2−^) and use S^0^, Fe^0^, S^2−^, and SO_4_^2−^ as electron donors to achieve nitrification denitrification and remove TN. Meanwhile, Acidobacteriota has also been widely detected in anaerobic ammonia oxidation processes [33]. The relative abundance of Acidobacteriota in ZJ.C.S is higher than that in QR.C.S, which can lead to a lower concentration of NH_4_^+^ in ZJ.C. However, Acidobacteriota accounts for less than 1% of the water in the treatment process of both eel farms. The high relative abundance of Cyanobacteria in QR.E.B is due to the fact that the concentration of NH_4_^+^, NO_3_^−^, NO_2_^−^, and TN in the water before entering the reaction ditch in QR farm is higher than that in the tailwater, leading to eutrophication of the water [34]. What is more, algae growth and photosynthesis limit the activity and abundance of nitrifying bacteria in aquaculture systems, weakening the ability of functional bacterial communities to remove NH_4_^+^ [35]. In general, high nutrient content (nitrogen and phosphorus) may lead to the dominance of nonfunctional bacteria, resulting in eutrophication. Eutrophication can enhance nitrogen fixation ability and weaken nitrogen removal ability of functional bacteria so that the nitrogen pollution content will further increase.

*Cetobacterium* is a common bacterial genus in the gut of freshwater fish [36], which accumulates with the discharge of aquaculture effluent. *Nitrospira* is a common nitrite-oxidizing bacterium (NOB) that can further oxidize NO_2_^−^ to NO_3_^−^ [37]. The nitrification process requires the synergistic action of ammonia-oxidizing bacteria (AOA/AOB) and NOB, but the current microbial community does not explicitly mention key nitrifying bacterial genera, such as *Nitrosomonas* or *Nitrococcus*, or bacterial genera that completely oxidize NH_4_^+^ to NO_3_^−^ [38]. If there is a lack of ammonia-oxidizing bacteria involved in the process of NH_4_^+^→ NO_2_^−^ in the early stage, the nitrification chain may be incomplete, which may lead to insufficient nitrification efficiency. Meanwhile, the presence of *Bdellovibrio* in both eel farms, a predatory bacterium, can also lead to a decrease in functional bacteria [39]. *Lactococcus*, a genus of fermenting bacteria abundant in both eel farms (Figure 4a), competes with other bacteria for carbon sources and inhibits the growth of functional bacteria [40]. The reduction in the number of functional bacterial genera or the inhibition of their activity will significantly weaken the ecological functions of functional bacterial communities in water bodies, such as the decline in the activity of key nitrogen conversion pathways, like nitrification and denitrification. This hinders the effective removal of nitrogen pollutants in water bodies through biogeochemical cycles mediated by indigenous microorganisms, leading to a decline in the efficiency of nitrogen pollution control.

In the current tailwater treatment process of aquaculture farms, nitrogen functional bacterial communities exhibit low abundance and single-function characteristics. What is worse, there are some genera that compete with nitrogen functional bacteria in water, and the synergistic effect between bacterial community structure and function is weak, ultimately leading to unsatisfactory nitrogen removal efficiency. So, to enhance nitrogen removal efficiency, multidimensional optimization strategies can be implemented through process intensification. On the microbial dimension, the structure and functional characteristics of the bacterial community can be improved by a targeted regulation of bacterial composition via the optimization of environmental parameters, coupled with bioaugmentation using high-efficiency nitrification functional strains, which enable functional enhancement of nitrogen metabolic pathways, etc. On the other hand, the filler or biofilm process can be used to expand the attachment space of functional bacteria and enhance the colonization stability and shock load resistance of bacteria in the system so as to improve the nitrogen removal efficiency and optimize the treatment process.

### 4.2. Pathogenic Microorganisms

In QR farm, *Vibrio* and *Edwardsiella* were detected only in QR.E.B, QR.D.S, and QR.C.B. While in ZJ farm, *Vibrio* and *Edwardsiella* appeared in the aquaculture pond and ZJ.F.S. Vibrio disease, as one of the common diseases in aquaculture, is caused by Gram-negative bacteria of the Vibrio family. This type of bacteria is widely present in aquatic environments and has opportunistic pathogenic characteristics towards fish and shellfish. When the disease occurs, infected aquatic animals often present with systemic infection symptoms, including external skin lesions, bleeding, and sepsis. In recent years, there have also been reports of cases of vibriosis in aquaculture farms in multiple countries [41]. In the aquaculture industry, *Edwardsiella* infection is an extremely common bacterial disease, especially in harsh aquatic environments, such as high temperatures and poor water quality, where its probability of occurrence significantly increases [42]. *Edwardsiella anguillarum* is a Gram-negative, rod-shaped, facultative anaerobic bacterium belonging to the genus *Edwardsiella*. It is commonly isolated from eels and is widely distributed in various aquatic environments [43,44]. Infected fish often exhibit symptoms of sepsis, accompanied by conditions such as eyeball protrusion, surface ulcers, and ascites. For eels, after infection, the liver will swell, and in severe cases, the liver and kidneys will develop ulcerative lesions, ultimately leading to a large number of deaths [45].

In recent years, the eel export restriction policy has led to a significant increase in the cost of aquaculture. Some farmers have turned to high-density, high-yield production models in pursuit of economic benefits, which has worsened the ecological environment of aquaculture ponds. The crowded environment for eel farming may also be conducive to the spread of pathogens [46]. At the same time, the issue of antibiotic resistance is becoming increasingly prominent, and the efficacy of previously used chemicals and antibiotics is gradually decreasing, further leading to a high incidence of diseases in eel farming [47]. That which occupies the highest proportion of the pathogens related to the aquaculture organisms in QR.C.S is *Lactococcus*, while that in QR.C.B is *Aeromonas*. *Lactococcus* can cause symptoms of sepsis in fish, and there have been cases in many countries where different fish species have been infected with *Lactococcus*, resulting in economic losses [48]. And, *Aeromonas* can cause bleeding, ulcer, decay, septicemia, and other diseases in fish (Table S2). The difference in the main pathogen between QR.C.S and QR.C.B indicates that the pathogen may be influenced by the form of the sample, with differences in environmental factors. Although the presence of some microbial species/strains belonging to known pathogen taxa may not necessarily have a negative impact on fish [49], such as *Pseudomonas,* whose relative abundance is high in both farms, if aquaculture farmers consider returning treated effluent to the aquaculture pond for recycling, they also need to pay attention to the hazards and prevention of pathogenic bacteria in aquaculture organisms in the water.

After being infected by pathogenic bacteria, aquaculture organisms accumulate toxins or act as carriers of pathogenic bacteria through the food chain, posing a threat to ecosystems and human health [50]. After consuming seafood infected with pathogenic bacteria, humans may experience adverse reactions such as gastroenteritis, diarrhea, and inflammation [51]. In addition, they can cause human eye, respiratory, skin, and soft tissue infections upon contact, wound exposure, or the use of contaminated water. In both farms, a substantial majority of the genera of pathogenic bacteria are identified as *Escherichia-Shigella* and *Legionella*. *Escherichia-Shigella* is a common pathogen of human digestive system diseases, which can cause diarrhea [52]. *Legionella* is a major opportunistic pathogen of concern in the drinking water system, and it is associated with pneumonia and respiratory diseases [53]. In ZJ.B.F, there is a unique genus of pathogenic bacteria, including *Neisseria*, which can possibly cause meningitis. It is mainly transmitted by respiratory droplets. So, it is necessary to remind aquaculture workers to strictly follow the requirements during the operation process. The enrichment of nutrients in the aquaculture wastewater treatment process leads to the proliferation of harmful algae, the degradation of the structure and function of surface water ecosystems, and the growth of microorganisms, including pathogenic bacteria in surface water. The overall surface water quality in the city is relatively high, but the effluent from ZJ farm and QR farm is not used as a water source but rather as a water source for agricultural and landscape use in Mayang River. It is noteworthy that the relative abundances of pathogenic bacteria at the discharge outlet of the two farms is the highest. If it needs to be used as a water source in the future, the risk of pathogenic bacteria must be controlled.

### 4.3. Correlation Analysis

As shown in Figure 5, environmental variables have an impact on the composition of microbial communities, and different samples exhibit community differentiation due to differences in environmental factors. At the phylum level, Desulfobacterota is negatively correlated with NO_3_^−^, NO_2_^−^, and TN, as most genera of Desulfobacterota exhibit the ability to utilize sulfides and hydrogen as electron donors, while NO_3_^−^ and NO_2_^−^ can act as acceptors for sulfate reduction and denitrification processes [54]. Planctomycetota is negatively correlated with NH_4_^+^, and studies have reported that Planctomycetota has anaerobic ammonia oxidation ability [55]. At the genus level, the *Bosea* genus is positively correlated with NO_3_^−^ and NO_2_^−^ and negatively correlated with NH_4_^+^, indicating that the *Bosea* genus may be involved in heterotrophic nitrification.

In water and sediment, pathogens associated with aquaculture organisms are mainly affected by NO_3_^−^ and NO_2_^−^, while pathogens related to humans are mainly affected by TN. But, in biofilms, NH_4_^+^ regulates the relative abundance of pathogenic bacteria. This implies that the concentration of pollutants has an impact on the composition of pathogenic bacteria. What is more, some pathogens are influenced by environmental media. They could be affected by different pollutants. *Escherichia-Shigella* is positively correlated with NO_3_^−^ in water and sediment and is positively correlated with NH_4_^+^ in biofilm. Or, it could be the opposite effect of the same environmental factors, like *Lactococcus,* which is positively correlated with NO_3_^−^ and NO_2_^−^ in biofilm but is the opposite in water and sediment. Therefore, for different sites or different forms of environmental samples, the prevention and treatment methods of pathogenic bacteria should be combined with the concentration of pollutants to make them more targeted. Furthermore, it is necessary to consider whether the pathogenic bacteria and dominant microorganisms caused by different pollutants are competitive with nitrogen functional bacteria.

**Figure 7:**
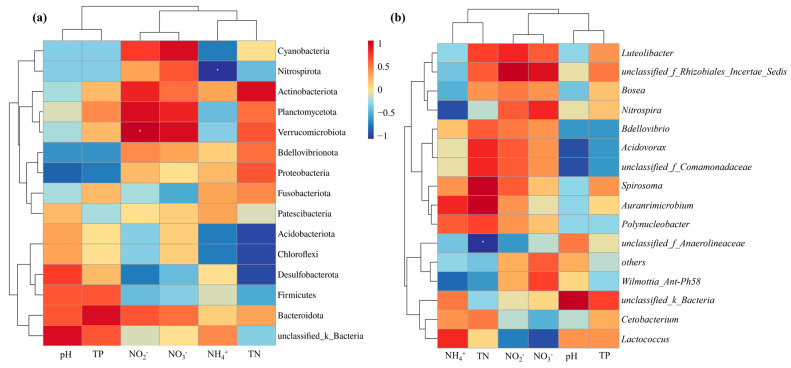
Correlation analysis between environmental factors and water and sediment microbial phylum and genus levels: (**a**) microbial phylum level in water and sediment; (**b**) microbial genus level in water bodies and sediment.

Combining the heatmap of water and sediment microbial phylum and genus levels with environmental factors (Figure 7), it can be seen that NO_2_^−^ is significantly positively correlated with Verrucomicrobiota (*p* < 0.05). NH_4_^+^ is significantly negatively correlated with Nitrospirota (*p* < 0.05). TN is negatively correlated with Chloroflexi, especially with *unclassified_f_Anaerolineaceae* (*p* < 0.05). Previous studies have shown that although most nitrification is a two-step process catalyzed by microorganisms to oxidize ammonia and nitrite, there are also a few strains belonging to *Nitrospira* that can perform complete nitrification on their own, activating both the genome-encoded pathways of ammonia oxidation and nitrite oxidation during the nitrification reaction [56]. *Unclassified_f_Anaerolineaceae* is a genus of bacteria belonging to the Anaerolineaceae family, which is a representative species of the Chloroflexi phylum and has the denitrification function and the ability to degrade carbohydrates and other cellular materials, such as amino acids [57]. However, it should be noted that some bacteria that are negatively correlated with NO_3_^−^, NO_2_^−^, and TN but are positively correlated with TP and pH, such as Desulfobacterota. So, if the introduction of nitrogen functional bacteria is adopted to promote the further denitrification of aquaculture tailwater, attention should be paid to whether the effect of nitrogen functional bacteria is limited by TP concentration and pH.

## 5. Conclusions

The nitrogen and phosphorus in the effluent from two freshwater eel farms in Nanping City exceed the discharge standards. The nitrogen pollutant in QR farm is mainly NH_4_^+^, while those in ZJ farm are mainly NO_3_^−^ and NO_2_^−^. Adding flocculants can effectively reduce phosphorus concentration, but there is no significant removal effect on nitrogen pollutants. In the current wastewater treatment process of aquaculture farms, the abundance of nitrogen functional bacteria is low, their functions are single, and the synergy of the bacterial community structure and function is poor, resulting in poor nitrogen removal efficiency. The environmental variables of the two farms have a significant impact on the composition of microbial communities, and different samples exhibit community differentiation due to differences in environmental factors. There are pathogenic bacteria present in the samples at each point. If the tailwater needs to be reused in the aquaculture pond, attention should be paid to the harm of pathogenic microorganisms to the aquaculture products, and if it is discharged into the water area that is a water source in the future, attention should be paid to the potential risks brought by pathogenic bacteria. What is more, the enrichment of pathogenic bacteria is influenced by environmental media or the concentrations of pollutants, which makes the attention paid to pathogens while controlling pollutants more important. This study put forward suggestions for the purification of aquaculture tailwater according to the analysis of the deficiencies of the current water treatment process and provide some support for the prevention of pathogen risk in aquaculture tailwater.

## Figures and Tables

**Table 1:** Water quality at different sites in two aquaculture farms.

Sample	Nitrogen and Phosphorus Pollutants (mg/L)					pH
	NH_4_^+^	NO_3_^−^	NO_2_^−^	TN	TP	
ZJ.B	0.06 ± 0.02	5.81 ± 0.20	1.930 ± 0.01	11.6 ± 0.18	0.98 ± 0.07	6.32 ± 0.05
ZJ.C	0.60 ± 0.03	3.81 ± 0.11	1.220 ± 0.01	6.16 ± 0.57	1.54 ± 0.02	7.39 ± 0.35
ZJ.D	0.67 ± 0.02	3.39 ± 0.23	1.154 ± 0.00	5.43 ± 0.48	1.29 ± 0.02	7.1 ± 0.01
QR.A	0.02 ± 0.01	0.25 ± 0.01	0.002 ± 0.00	0.33 ± 0.05	0.07 ± 0.00	6.86 ± 0.04
QR.B	9.81 ± 0.07	3.50 ± 0.11	1.388 ± 0.01	15.63 ± 1.15	2.00 ± 0.01	7.26 ± 0.02
QR.C	3.98 ± 0.12	1.89 ± 0.29	0.439 ± 0.00	6.74 ± 0.11	1.27 ± 0.03	6.84 ± 0.01
QR.F	5.36 ± 0.07	2.93 ± 0.07	0.762 ± 0.00	9.03 ± 0.69	0.04 ± 0.00	6.7 ± 0.03

## Data Availability

The original contributions presented in this study are included in the article/Supplementary Material. Further inquiries can be directed to the corresponding authors.

## Supplementary Materials

The following supporting information can be downloaded at https://www.mdpi.com/article/10.3390/microorganisms13071624/s1. Table S1: Requirement for water discharge from freshwater aquaculture pond (SC/T 9101-2007). Table S2: Some pathogenic bacteria and symptoms of infection in aquaculture. References [58,59,60,61,62,63,64,65,66,67,68,69,70,71] are cited in the supplementary materials.
