# Supplementary material for: Characterization, Microbial Community Structure, and Pathogen Occurrence in Two Typical Eel Farms
Source: Microorganisms. 2025 Jul 10;13(7):1624. doi: 10.3390/microorganisms13071624 (PMC12298136; doi:10.3390/microorganisms13071624)
Supplement: Supplementary file 1 [file microorganisms-13-01624-s001.zip › microorganisms-3717727-supplementary.pdf]

**Table S1:** Requirement for water discharge from freshwater aquaculture pond (SC/T 9101-2007)

| Number | Item | First-class standard （mg/L） | Second-class standard（mg/L） |
|---|---|---|---|
| 1 | Suspended substance | ≤50 | ≤100 |
| 2 | pH | 6.0～9.0 | |
| 3 | $COD_{Mn}$ | ≤15 | ≤25 |
| 4 | $BOD_5$ | ≤10 | ≤15 |
| 5 | Zn | ≤0.5 | ≤1.0 |
| 6 | Cu | ≤0.1 | ≤0.2 |
| 7 | TP | ≤0.5 | ≤1.0 |
| 8 | TN | ≤3.0 | ≤5.0 |
| 9 | Sulfide | ≤0.2 | ≤0.5 |
| 10 | Total residual chlorine | ≤0.1 | ≤0.2 |

**Table S2:** Some pathogenic bacteria and symptoms of infection in aquaculture

| Pathogenic bacteria | Symptoms of infection | References |
|---|---|---|
| *Edwardsiella anguillarum* | Septicemia, exophthalmos, body surface ulcers, ascites, hepatomegaly, etc. | [58] |
| *Chryseobacterium balustinum* | Fin erosion, eye, surface bleeding, exophthalmos, organ liquefaction | [59] |
| *Acinetobacter pittii* | Ulcerative lesions, bleeding, septicemia of skin and caudal fin decay | [60] |
| *Pseudomonas anguilliseptica* | Body surface and visceral bleeding, splenomegaly, hemorrhagic renal swelling, ulcer, etc. | [61] |
| *Streptococcus iniae* | Exophthalmos, lethargy, back stiffness, meningoencephalitis, ulcer, sepsis, ascites, etc. | [62] |
| *Vibrio anguillarum* | Surface ulcer, congestion in gills, fins, kidneys, spleen and other parts, liver enlargement, abdominal effusion | [63] |
| *Lactococcus garvieae* | Bleeding from fins, liver, kidney and spleen | [64] |
| *Aeromonas hydrophila* | Bleeding, ulcer, decay, septicemia | [65] |
| *Cytophaga columnaris* | Yellow lesions on gill, skin and fin of fish | [66] |
| *Enterococcus faecalis* | Ulcer and inflammation | [67] |
| *Flavobacterium psychrophilum* | Skin injury and gill necrosis | [68] |
| *Francisella noatunensis* | Splenomegaly and nephromegaly | [69] |
| *Nocardia brasiliensis* | Hemorrhagic ascites, organ enlargement, epidermal abscess and nodules in gills, kidneys and spleen | [70] |
| *Mycobacterium fortuitum* | Skin ulcer, inflammation, liver and kidney granuloma and necrosis, intestinal bleeding | [71] |
